# Exploring the Correlation Between Interatomic Bonding and Corrosion Resistance of Metallic Alloys via Combinatorial Method

**DOI:** 10.1002/advs.202504168

**Published:** 2025-05-11

**Authors:** Liwei Hu, Fucheng Li, Weijie Xie, Chao Wang, Mingxing Li, Gang Wang, Yanhui Liu

**Affiliations:** ^1^ Institute of physics Chinese Academy of Science Beijing 100190 China; ^2^ School of Materials Science and Engineering Shanghai University Shanghai 200444 China; ^3^ Center of Materials Science and Optoelectronics Engineering University of Chinese Academy of Sciences Beijing 100049 China

**Keywords:** combinatorial method, corrosion resistance, element characteristics, metallic glass

## Abstract

Developing metallic alloys with excellent corrosion resistance is of great significance for ensuring the long‐term integrity and reliability of materials in various demanding environments, thereby extending their service life and reducing maintenance costs. However, the corrosion of alloys is a complicated process influenced by many factors, such as composition, structure and surface finishing, and corrosion media. Current evaluations of alloy corrosion resistance involve many steps, which are time‐consuming and laborious to explore within a vast compositional space. In this study, 1874 alloys from 8 alloy systems are prepared and characterized using a combinatorial approach. Analyses of the data indicate that corrosion resistance of an alloy is strongly correlated with metal–metal bond strength (ε_M–M_) and metal–oxygen bond strength (ε_M–O_). Enhanced corrosion resistance can be achieved by alloying elements with high ε_M–M_ and ε_M–O_. The consideration from interatomic interactions further reveals that adding elements with high ε_M–M_ and ε_M–O_ to a base alloy system actually lowers the critical weight‐averaged ε_M–M_ and ε_M–O_ required for corrosion resistance. The ε_M–M_ and ε_M–O_ guided selection of alloying elements is applicable in different alloy systems. This finding will facilitate the fast discovery of novel alloys with superior corrosion resistance.

## Graphical abstract

1874 alloys from 8 alloy systems are prepared and characterized using a combinatorial approach. Analyses into the data indicate that corrosion resistance of an alloy is strongly correlated with metal–metal bond strength (ε_M–M_) and metal–oxygen bond strength (ε_M–O_). The consideration from interatomic interactions further reveals that adding elements with high ε_M–M_ and ε_M–O_ to a base alloy system actually lowers the critical weight‐averaged ε_M‐M_ and ε_M‐O_ required for corrosion resistance.

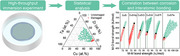

## 1 Introduction

Corrosion of metallic components can significantly damage engineering structures and cause premature failures of their functions, resulting in substantial economic losses and societal impact.^[^ ^1^ ^]^ Enhancing the durability of metallic components requires the efficient design of corrosion‐resistant alloys.^[^ ^2^ ^]^ Generally, the corrosion resistance of metallic alloys can be improved either by alloying or tailoring their microstructure through heat treatment,^[^ ^3^, ^4^ ^]^ structural amorphization,^[^ ^5^, ^6^ ^]^ and surface finishing^[^ ^7^, ^8^ ^]^ et al. Among these approaches, alloying beneficial elements is a simple yet effective method. The alloying effects include increased density and reparability of the passive film, reduced localized corrosion, microstructure refinement, and formation of corrosion‐resistant phases.^[^ ^9^, ^10^, ^11^, ^12^ ^]^ For example, adding Ni to duplex stainless steels leads to refined microstructure and Ni enrichment in the passive film, thereby enhancing the pitting potential.^[^ ^11^ ^]^ Similarly, when the Cr content in Ni and Co exceeds a critical threshold, a dense passive film forms, which tightly adheres to the substrate and substantially reduces the passive current density.^[^ ^9^ ^]^ However, selecting appropriate alloying elements and determining their optimal concentrations is challenging because of the complex interactions among the constituent components.

According to Marcus's proposal, the alloyed elements can be divided into two categories based on their functions. The passivity promoters are elements that combine a high metal‐oxygen bond strength with a low metal–metal bond strength. These elements promote the formation of stable passive films. In contrast, the dissolution moderators have a high metal–metal bond strength, which increases the activation energy barrier for the disruption of metal–metal bonds at the surface, and thus decelerates dissolution from the matrix.^[^ ^13^, ^14^ ^]^ Nonetheless, it should be noted that whether an element serves as a passivity promoter or dissolution moderator is dependent on the base alloy. Adding the same element into different alloy systems does not always have a positive effect on the corrosion behavior. For example, adding Ni element to the Ti_41_Zr_25_Be_34_ glass‐forming alloy promotes the formation of denser and more stable passive film.^[^ ^15^ ^]^ However, Ni deteriorates the corrosion resistance in the Ir‐Ni‐Ta glass‐forming alloy system.^[^ ^6^ ^]^ Therefore, it remains elusive what elements are effective for improved corrosion resistance for a specific alloy system. The effects of metal–metal bonding and metal‐oxygen bonding have successfully guided the design of efficient catalysts.^[^ ^16^ ^]^ However, their role in the design of corrosion‐resistant alloys has not yet received sufficient attention. In addition, the concentration of alloying elements should also be considered not only due to the need for phase stability, desirable mechanical properties, and minimization of cost,^[^ ^17^ ^]^ but also due to the fact that there may exist a threshold concentration below which the beneficial effects of alloying cannot be achieved.^[^ ^9^ ^]^

Multicomponent crystalline alloys often undergo compositional redistribution and the precipitation of different phases. The variations in the types, sizes, and distributions of these phases have distinct effects on the corrosion behavior of the alloys.^[^ ^18^, ^19^ ^]^ Therefore, it is challenging to precisely investigate the compositional effect on corrosion behavior through crystalline alloys. Metallic glasses provide the platform for examining the effect of compositions on corrosion resistance. It is known that metallic glasses are free of grain boundaries and second phases. The amorphous structure can be chemically homogenous down to the atomic scale.^[^ ^20^ ^]^ Therefore, the effect of microstructures can be neglected, allowing a more precise investigation of the relationship between composition and corrosion properties. In addition, metallic glasses have obtained widespread attention due to their superior corrosion resistance^[^ ^21^ ^]^ and represent an important class of corrosion‐resistant alloys. To date, numerous studies have been conducted on the evolution of the corrosion performance of metallic glasses with compositional variations.^[^ ^6^, ^20^, ^22^, ^23^ ^]^ Nevertheless, predicting how the evolution of corrosion properties changes with chemical composition remains a challenge. Therefore, there is an urgent need to develop efficient guidance to identify how the additions of metallic elements enhance the corrosion resistance of materials.

In this work, we take metallic glasses as model materials to develop a straightforward yet effective method to investigate the alloying effect on the corrosion resistance of metallic alloys. Specifically, we employ combinatorial methods to fabricate and characterize 1374 based on Cu–Zr alloy system. Cu–Zr was selected because it is the base system from which many commercial bulk metallic glasses with superior properties, such as Vit1, Vit105, and Vit106, were discovered and optimized.^[^ ^24^ ^]^ We add different elements to Cu–Zr and statistically analyze the changes of the critical concentration above which the materials can withstand the attack of corrosive media. The result indicates a strong correlation between the corrosion resistance and metal–metal bond strength (ε_M–M_) and metal–oxygen bond strength (ε_M–O_). It turns out that adding elements with higher ε_M‐M_ and ε_M–O_ can enhance the critical concentration of Cu, thereby improving corrosion resistance. This work provides ε_M–M_ and ε_M–O_ guided selection of alloying elements for the improvement of corrosion resistance and suggests that these parameters can be fundamental descriptors for the prediction of new alloys by machine learning approaches.

## 2 Results and Discussion

**Figure 1:**
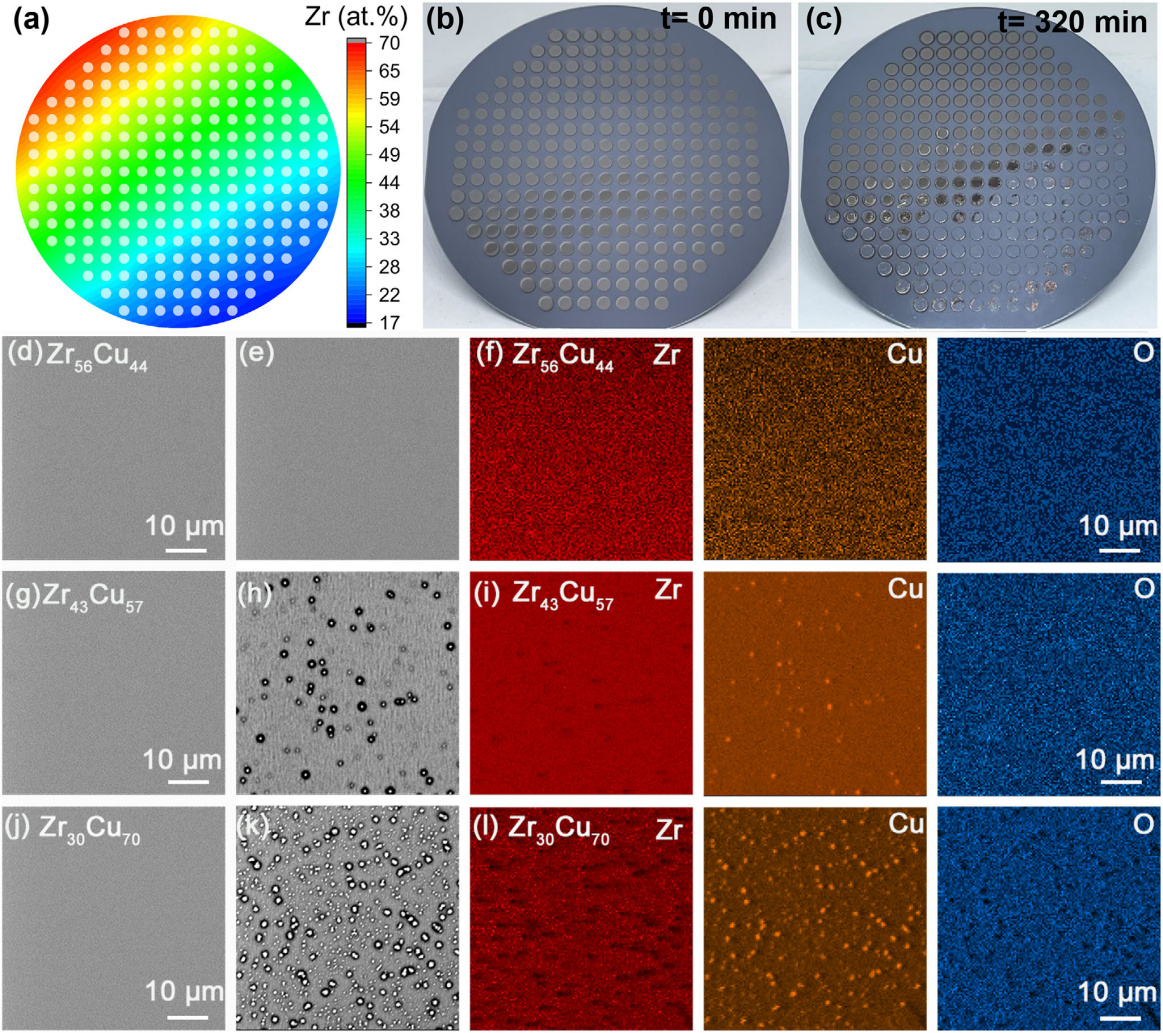
Compositional characterization of Cu‐Zr alloy library and its surface images before and after corrosion measurement in 3.5 wt.% NaCl solutions at 298 K for 320 min. a) Counter plots for the concentration of Zr on a diameter of 100 mm Si wafer. The physical positions of the alloys are labeled as circles. b,c) Appearance of the Zr–Cu compositional library immersed in 3.5 wt.% NaCl solution at 298 K for the labeled immersion times. d–l) SEM images and EDS mapping results of the before and after corrosion for the three alloys of Zr_56_Cu_44_, Zr_43_Cu_57_, and Zr_30_Cu_70_.

The combinatorial alloy library is created by using magnetron co‐sputtering deposition with which the composition gradient of the library can be varied by tuning the sputter power applied on targets.^[^ ^26^ ^]^ As shown in **Figure** **1**a,b, each library contains 229 alloy patches with a diameter of 3.6 mm. The thickness of the film ranges from 800 to 1000 nm. Chemical analysis by EDX on each alloy reveals that the composition variation within the binary Cu–Zr library ranges from 34 to 80 at.% for Cu and from 20 to 66 at.% for Zr. The average composition variations within each patch is ≈2.5 at.%. XRD mapping indicates that all the Cu–Zr alloys exhibit an amorphous structure, which is consistent with previous investigations.^[^ ^27^ ^]^ To reveal the variation of corrosion resistance with composition in the Cu–Zr binary system, the library is immersed in 3.5 wt.% NaCl solution at 298 K. Figure 1b,c show the appearance of the library before and after immersion for 320 min. It can be seen that the Cu–rich alloys exhibit significant changes in surface color. Some patches are even damaged by the immersion and detach from the substrate. To disclose the mechanism accounting for the corrosion of the Cu–rich alloys, we analyze three alloys of different Cu content (Zr_56_Cu_44_, Zr_43_Cu_57,_ and Zr_30_Cu_70_). The changes in surface morphologies after 320 min of immersion are presented in Figure 1d,e,g,h,j,k for the three alloys. Initially, the surfaces of the as‐prepared alloy patches appear smooth and featureless. After immersion in NaCl solution, corrosion products covering the surfaces are observed. EDX mapping on the corroded surface reveals that the particle‐like corrosion products are Cu–rich oxides (see Figure 1f,i,l). This indicates that when Zr passivation layers fail to form effectively, the corrosion of the Zr–Cu alloy originates from the dissolution of Cu from the surface.^[^ ^28^ ^]^ However, the number density of the corrosion products varies significantly among the alloys. The densest corrosion product is found in Zr_30_Cu_70_, the alloy containing the highest Cu content among the three alloys. In contrast, the surface of Zr_56_Cu_44_ is nearly free of corrosion products. The difference in the number density of Cu–rich oxides on the alloys highlights the critical role of Cu concentration on corrosion resistance, with Cu–lean alloys being more corrosion resistant. From the boundary separating damaged and undamaged regions, the critical Cu concentration ($C_{Cu}^{BD}$) is determined to be 51.6 ± 3.3 at.%. This critical concentration, $C_{Cu}^{BD}$, provides an essential reference for evaluating the effect of alloying element on corrosion resistance.

**Figure 2:**
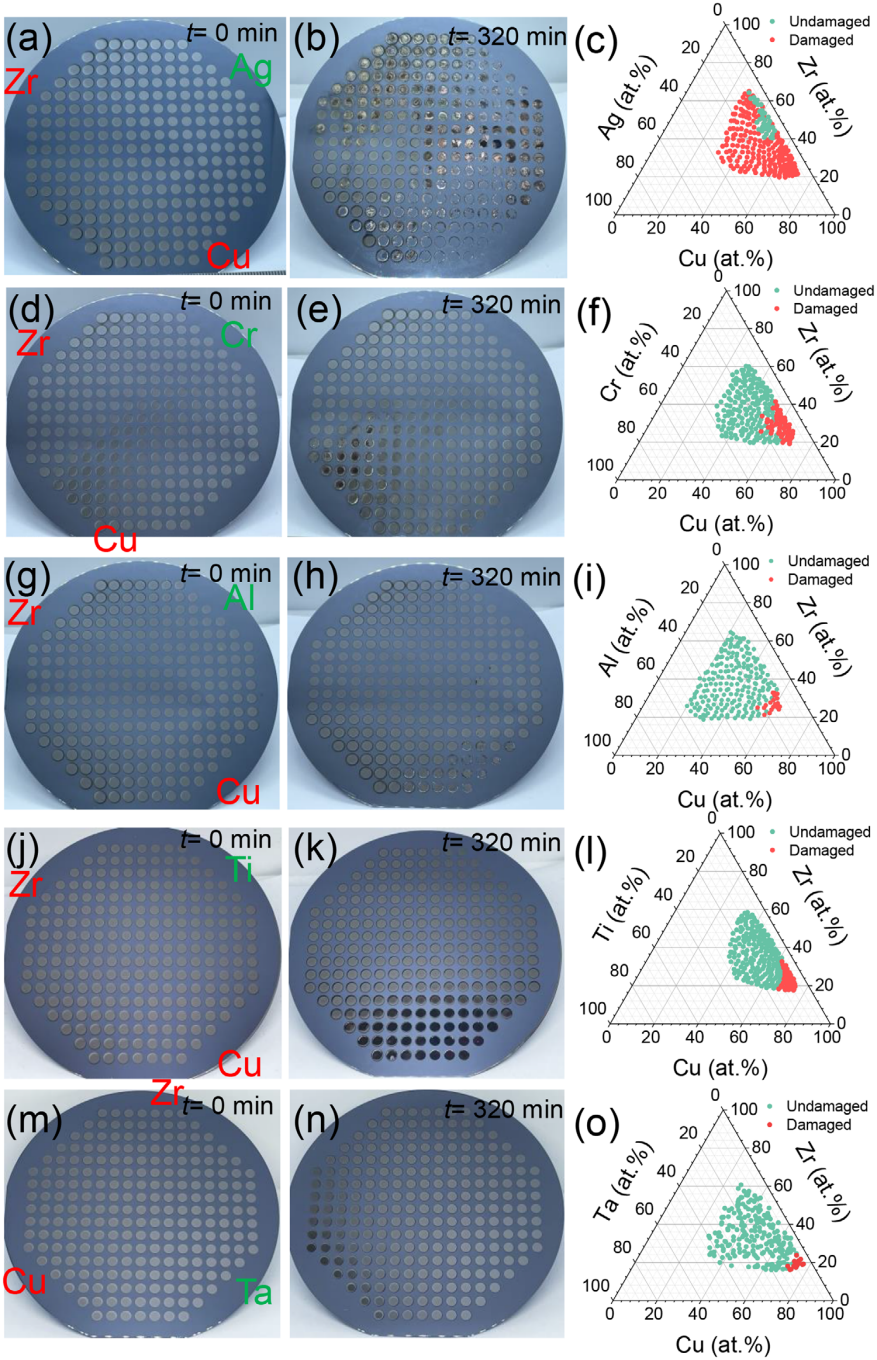
Compositional characterization and visual appearance of the Cu–Zr–X (X: Ag, Cr, Al, Ti, and Ta) compositional libraries before and after 320 min of immersion in 3.5 wt.% NaCl solutions at 298 K. a–c) Cu–Zr–Ag alloy system, d–f) Cu–Zr–Cr alloy system, g–i) Cu–Zr–Al alloy system, j–l) Cu–Zr–Ti alloy system, m–o) Cu–Zr–Ta alloy system. In each alloy system, the green dots represent the composition of the undamaged alloy after immersion, while the red dots represent damaged alloys.

We incorporate Ag, Cr, Al, Ti, and Ta into the binary Zr–Cu system. These five elements are commonly used for improving the corrosion resistance of metallic alloys. We investigate how $C_{Cu}^{BD}$ varies relative to the reference concentration (51.6 ± 3.3 at.%) with the addition of these elements. This allows for revealing the intrinsic relationship between the effect of alloying elements and the corrosion resistance, thus providing potential guidelines for the design of corrosion‐resistant alloys. We create combinatorial alloy libraries for these ternary systems and immerse them in a 3.5 wt.% NaCl solution. Similar to the Zr–Cu library, each ternary library contains 229 alloy patches. By studying a total of 1145 alloys, we observe the alloying‐induced variation of corrosion resistance. **Figure** **2** shows the appearance of all ternary combinatorial libraries before and after 320 min of immersion in the 3.5 wt.% NaCl solution. As shown in Figure 2, significant changes of surface color, and in some cases complete destruction of patches due to corrosion, are observed in the Cu–rich regions of each library. In contrast, little changes are seen on the Zr‐rich regions. These differences in corrosion resistance are consistent with the case in the binary Zr–Cu library and previous studies on similar amorphous alloys.^[^ ^29^, ^30^, ^31^ ^]^ Zr tends to form a dense ZrO_2_ film on the surface, preventing rapid dissolution of the matrix, while Cu tends to form porous Cu–Cl films in chloride‐containing solutions. The Cu–Cl films eventually evolve into Cu_2_O through hydrolysis, which cannot prevent the dissolution of metals from the surface.^[^ ^30^ ^]^ As a result, the Cu–rich regions exhibit much lower corrosion resistance. It is noteworthy that the addition of different elements to the binary Zr–Cu results in distinct variations in corrosion behavior. For example, almost all alloy patches in the library with Ag addition are destroyed (Figure 2c). This indicates that the addition of Ag leads to a reduction in corrosion resistance. This is consistence with previous reports that the addition of Ag is detrimental to the corrosion resistance of Zr‐based metallic glasses.^[^ ^32^, ^33^ ^]^ In contrast, few alloy patches in the library with Ta addition detach from the substrate, except for some changes in surface color in a narrow Cu–rich region (Figure 2o). Although Al and Cr are often considered effective alloying elements for enhancing corrosion resistance, patch detachment is observed in the libraries with Al (Figure 2i) and Cr (Figure 2f) addition. The effect of Ti addition appears to lie in between that of Ta and Al: the color change region is larger than that in the library with Ta addition, but the number of detached patches is fewer than in the library with Al addition. A more quantitative comparison is provided in the triangle plots, where the destroyed patches are marked in red.

**Figure 3:**
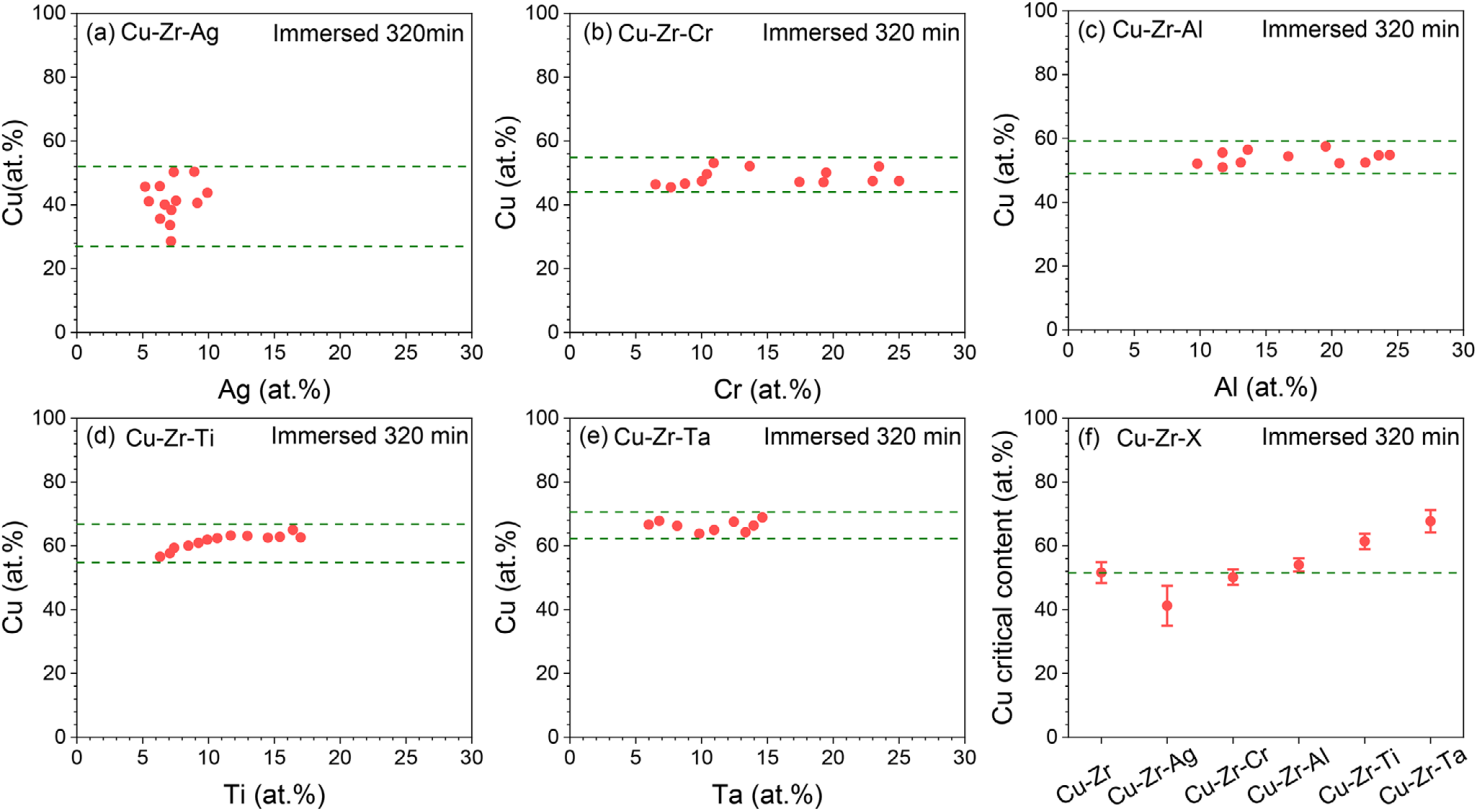
Cu Content threshold at undamaged boundaries in Cu–Zr–X combinatorial materials library after immersed in 3.5 wt.% NaCl solution for 320 min. a) Cu–Zr–Ag, b) Cu–Zr–Cr, c) Cu–Zr–Al, d) Cu–Zr–Ti, e) Cu–Zr–Ta, f) Comparison of Cu content threshold for all alloy libraries.

To further analyze the effect of alloying elements on the corrosion resistance of Zr–Cu system, we carefully identify the boundary between damaged and undamaged patches and plot $C_{Cu}^{BD}$ as a function of the amount of alloying element added (**Figure** **3**). By comparing the variation of $C_{Cu}^{BD}$ with the addition of different alloying elements, we can assess their influence on the corrosion resistance of the Cu–Zr alloys. Specifically, if $C_{Cu}^{BD}$ exceeds the reference concentration (51.6 ± 3.3 at.%) with the addition of an alloying element, it indicates that the element prevents Cu dissolution, thereby allowing more Cu to remain in the alloy. This suggests that the element enhances the corrosion resistance of the base alloy and vice versa. As can be seen in Figure 3a, with the addition of Ag, $C_{Cu}^{BD}$ decreases significantly from the reference concentration of 51.6 ± 3.3 to 41.2 ± 6.3 at.%, indicating that Ag addition actually reduces the corrosion resistance of Zr–Cu alloys, consistent with the observed patch detachment in the library after immersion. Although Cr and Al have been frequently used as alloying elements in the design of corrosion‐resistant alloys, the addition of Cr (Figure 3b) and Al (Figure 3c) results in no significant change in $C_{Cu}^{BD}$ relative to the reference concentration. For example, the addition of Cr results in $C_{Cu}^{BD}$ of 50.1 ± 2.4 at.%, while the concentration for Al addition is 54 ± 2.1 at.%. This suggests that Cr and Al essentially play a neutral role in the Zr–Cu alloys. In contrast, the addition of Ti (Figure 3d) and Ta (Figure 3e) raises $C_{Cu}^{BD}$ to much higher values (61.4 ± 2.3 at.% and 67.7 ± 3.5 at.%, respectively) compared to reference concentration, suggesting their effectiveness in enhancing the corrosion resistance of Zr–Cu alloys. Figure 3f summarizes $C_{Cu}^{BD}$ for all of the considered alloy systems. One can see that the order of enhancing the corrosion resistance of Zr–Cu alloys is Ta>Ti>Al>Cr>Ag.

**Figure 4:**
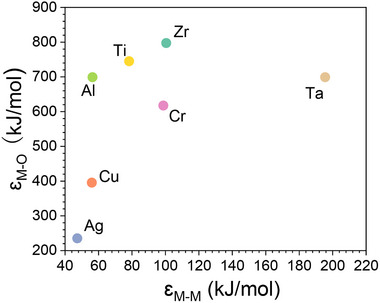
The values of metal–metal bond strength (ε_M–M_) and metal‐oxygen bond strength (ε_M–O_) for the corresponding metallic elements that are related to corrosion performance. ε_M–M_ is estimated according to the weighted average of the heat of sublimation and the coordination number of the metal. ε_M–O_ is estimated according to the weighted average of the heat of the formation of metallic oxide.^[^ ^13^ ^]^

According to Marcus, the ability of an element to enhance corrosion resistance of the base alloy is related to both the metal–metal bond strength (ε_M–M_) and metal‐oxygen bond strength (ε_M–O_) of the element.^[^ ^21^, ^34^ ^]^ The ε_M–M_ reflects the resistance of an element to dissolution from the surface, while the ε_M–O_ determines its ability to form a stable oxide passive film.^[^ ^21^, ^34^ ^]^ Our previous works confirmed that corrosion resistance improves with increasing strength of both ε_M–M_ and ε_M–O_.^[^ ^6^ ^]^ The ε_M‐M_ values for Cu, Zr, Ag, Cr, Al, Ti, and Ta are 56, 100.5, 47.3, 98.75, 56.4, 78.3, and 195.5 kJ mol^−1^, respectively, while the corresponding ε_M‐O_ values are 395.5, 797.3, 235.2, 617.5, 699, 745, and 699 kJ mol^−1^, respectively (**Figure** **4**). It is evident that both ε_M‐M_ and ε_M‐O_ for Ag are much lower than those for Cu and Zr. The weak metal–metal bond strength of Ag, along with its low metal‐oxygen bond strength, suggests that Ag can neither prevent metal dissolution nor facilitate the formation of stable passive film in NaCl solution. Instead, Ag is more likely to be preferentially dissolved compared to Cu and Zr during corrosion, thereby reducing the corrosion resistance. Cr and Al are considered neutral elements regarding the corrosion resistance of Zr–Cu, because $C_{Cu}^{BD}$ is essentially unchanged (50.1 ± 2.4 at.% and 54 ± 2.1 at.% for Cr and Al, respectively) relative to the reference concentration (51.6 ± 3.3 at.%). This neutral role of Cr and Al can be attributed to the combined effects of ε_M–M_ and ε_M–O_. Specifically, the ε_M–M_ value of Al is smaller than Cu, but its ε_M–O_ is larger. This implies that Al may be beneficial for the formation of stable passive film, but the small ε_M–M_ cannot prevent rapid dissolution metallic ions and thus decreasing corrosion resistance.^[^ ^35^ ^]^ Cr is slightly less effective than Al in increasing $C_{Cu}^{BD}$. This can be ascribed to the smaller ε_M–O_ of Cr than Al. Although the ε_M–M_ of Cr is larger than Al and thus enhances the activation barrier for dissolution, the smaller ε_M–O_ decreases its ability to form an effective passivation film during the corrosion process. Ti exhibits the strongest ε_M–O_ and a higher ε_M–M_ than Cu. These suggest that the addition of Ti facilitates the early formation of a stable passivation film preventing further corrosion of the matrix,^[^ ^15^ ^]^ and thus leading to increased $C_{Cu}^{BD}$. The analysis on the effect of Ag, Cr, Al, and Ti suggests that to improve corrosion resistance, a balance must be maintained among ε_M–M_ and ε_M–O_. As seen in Figure 4, Ta has the highest ε_M–M_ and a considerable ε_M–O_. These values indicate that Ta is effective in increasing the activation energy barrier for dissolution from the surface and promotes the formation of stable passive film, resulting in a significant increase of $C_{Cu}^{BD}$ and improved corrosion resistance of Zr–Cu binary alloys.

Although the above analyses based Marcus's proposal can explain the role of each element in the corrosion resistance of the Zr–Cu system, interatomic interactions are not considered. As a first approximation, we calculate the weight‐averaged ε_M–M_ and ε_M–O_ values, which account for the interactions of all constituent elements.^[^ ^6^ ^]^ The metal–metal bond strength (ε_M–M_) can be expressed as:

(1) $$\varepsilon_{M-M}=\frac{\sum x_{i}\cdot x_{j}\cdot \left[\Delta H_{i,j}/\left(\frac{Z_{i,j}}{2}\right)\right]}{\sum x_{i}\cdot x_{j}}$$

where Δ*H_i,j_* is the heat of sublimation, Z is the coordination number. *x_i_* and *x_j_* represents the atomic percentage of the *i*‐th and *j*‐th constituent element in the alloy composition, respectively.^[^ ^36^, ^37^ ^]^ The metal‐oxygen bond strength ε_M–O_, is related to the heat of adsorption of oxygen by the following relation:

(2) $$\varepsilon_{M-O}=\left\{\sum \left[x_{i}\cdot \Delta H_{i,\;ads}\left(ox\right)\right]+D\left(O_{2}\right)\right\}/2$$

where *D*(O_2_) is the dissociation energy of O_2_ (498 kJ mol^−1^) and ΔH*_i,ads_* (ox) is the heat of adsorption of oxygen.^[^ ^38^, ^39^ ^]^

**Figure 5:**
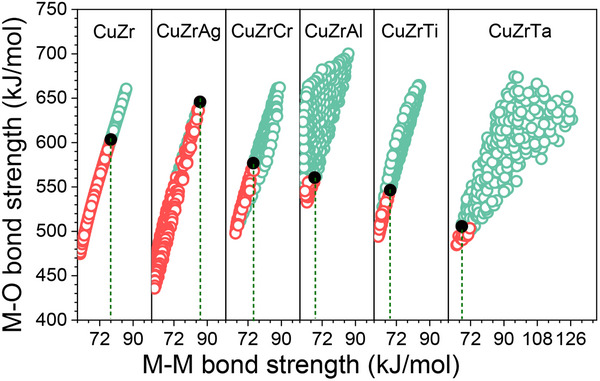
The average metal–metal bond strength and metal–oxygen bond strength for 1374 alloys from six compositional materials libraries based on Cu–Zr system. The red dots represent the damaged alloys after immersion 320 min in 3.5 wt.% NaCl solutions and the green dots represent the undamaged alloys in each alloy library. The black dots indicate the threshold ε_M–M_ and ε_M–O_ of undamaged alloy thin film in the corresponding alloy systems.

The calculated ε_M–M_ and ε_M–O_ values for the binary Zr–Cu system and all ternary alloy systems are shown in **Figure** **5**. It is worth noting that for each alloy system, there exist critical ε_M–M_ and ε_M–O_ values above which the alloy patches remain intact after immersion. For Cu–Zr alloy system, the critical values of ε_M–M_ and ε_M–O_ are 78 and 603 kJ mol^−1^, respectively. After elements addition, the critical values of ε_M‐M_ for alloy libraries of Cu–Zr–Ag, Cu–Zr–Cr, Cu–Zr–Al, Cu–Zr–Ti and Cu–Zr–Ta are 86, 74.8, 68, 68.7, and 67.2 kJ mol^−1^, respectively. The corresponding ε_M‐O_ for these alloy libraries are 645.9, 576.9, 560.6, 546.4, and 505.6 kJ mol^−1^, respectively. One can see that the critical values of ε_M–M_ and ε_M–O_ decrease when the elements are added to the Cu–Zr binary system except for the addition of Ag. The higher the ε_M–M_ and ε_M–O_ of the added elements have, the lower the critical ε_M‐M_ and ε_M–O_ become. The decreased critical ε_M–M_ and ε_M–O_ reflects the increased $C_{Cu}^{BD}$. For example, in the Cu–Zr–Ag alloy library, the alloy corresponding to the critical ε_M–M_ and ε_M–O_ values is Cu_28_Zr_65_Ag_7_. As noted above, the decreased $C_{Cu}^{BD}$ manifests that the addition of Ag deteriorate corrosion resistance of Cu–Zr alloys. Therefore, a higher content of Zr is necessary to compensate the Ag‐addition‐induced decreases in ε_M–M_ and ε_M–O_, because the ε_M–M_ and ε_M–O_ of Zr are higher than both that of Cu and Ag. This ultimately results in the increased critical ε_M–M_ and ε_M–O_ values of the Cu–Zr–Ag alloy library compared to that of the Cu–Zr binary alloy library. In the Cu–Zr‐Ta alloy library, the alloy corresponding to the critical ε_M–M_ and ε_M–O_ is Cu_71_Zr_24_Ta_5_. As shown in Figure 4, Ta is of high ε_M–M_ and ε_M–O_, its addition to Cu–Zr can reduce the dissolution rate and promote the formation of stable passive film, and thus, a small amount of Ta addition is able to protect the Cu–Zr alloys from being damaged during immersion even if the Cu–Zr alloys contain high concentration of Cu. The high $C_{Cu}^{BD}$ leads to a decreased critical ε_M–M_ and ε_M–O_ values in the Cu–Zr–Ta library. The arguments suggest that lower critical ε_M–M_ and ε_M–O_ at $C_{Cu}^{BD}$ is beneficial for enhanced corrosion resistance because $C_{Cu}^{BD}$ can be increased.

The observations on the variation of $C_{Cu}^{BD}$ upon addition of different elements implies two rules for the selection of elements to enhance the corrosion resistance of the Cu–Zr alloy. On the one hand, elements with ε_M–M_ close to that of Cu and higher ε_M–O_ value, such as Ti, should be selected to promote the formation of a stable passive film. Second, elements with much higher ε_M–M_ value than that of Cu, such as Ta, should be selected to hinder the dissolution of metallic ion from the surface.

**Figure 6:**
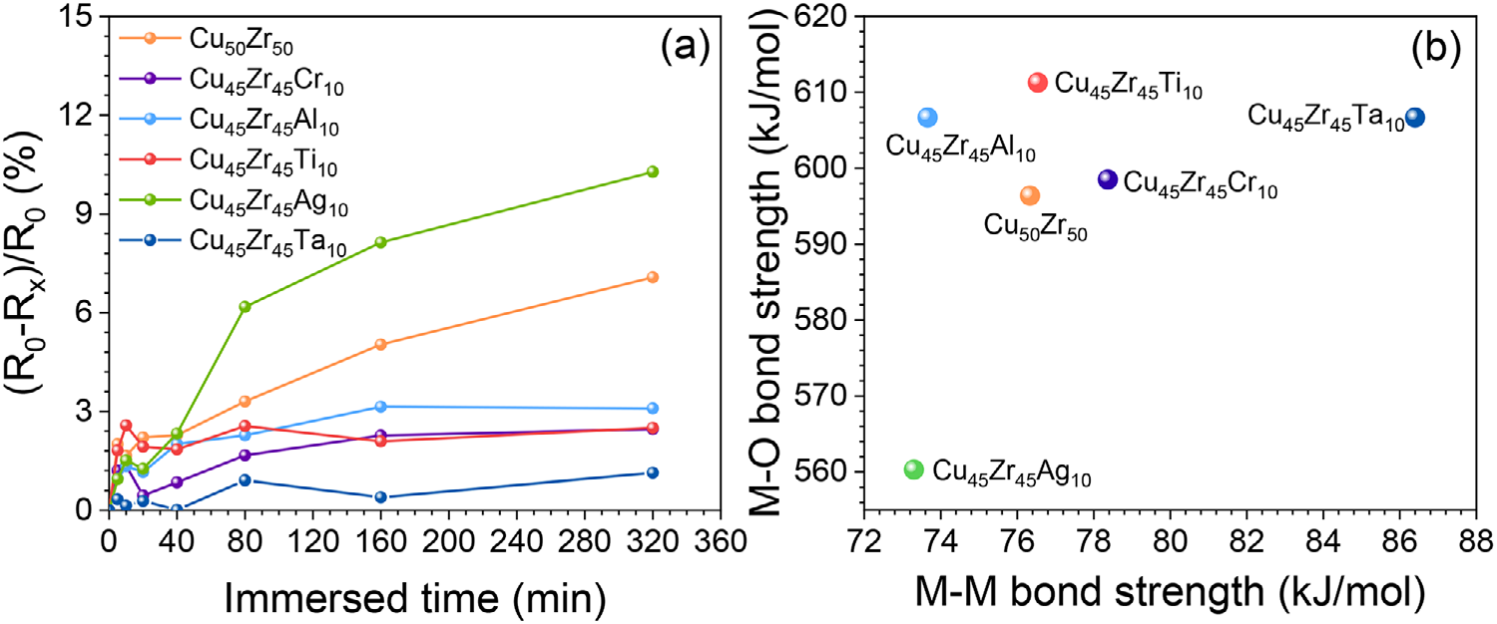
The relationship between corrosion resistance of representative alloys and their ε_M–M_ as well as ε_M–O_. a) Reflectivity changes of the representative alloy thin films immersed in 3.5 wt.% NaCl solution at different times. R_0_ represents the reflectivity of alloy thin films without immersion, R_x_ represents the reflectivity of alloy thin films after immersion at selected times. b) The average ε_M–M_ and ε_M–O_ for corresponding alloys.

To further demonstrate the influence of ε_M–M_ and ε_M–O_ on the corrosion behavior of Cu–Zr alloys, we compare the corrosion resistance of alloys after adding different elements. Specifically, we prepared Cu_45_Zr_45_X_10_ thin films (X = Ag, Cr, Al, Ti, and Ta) and measured the variation of surface reflectance of the films after immersion in 3.5 wt.% NaCl solution. According to the literature, the changes of optical constants reflect the degree of corrosion or oxidation.^[^ ^20^, ^40^ ^]^ For example, a larger change in reflectance in the Cu–Zr–Al alloy system indicates higher corrosion current density and weaker corrosion resistance.^[^ ^20^ ^]^ **Figure** **6**a displays the change of reflectivity as a function of immersion times for the Cu_45_Zr_45_X_10_ thin films. One can see that the reflectivity changes of the Cu_45_Zr_45_X_10_ films are smaller than the Cu_50_Zr_50_ alloy, except for Cu_45_Zr_45_Ag_10_. These indicate that the addition of Cr, Al, Ti, and Ta enhances the corrosion resistance of Cu_50_Zr_50_ alloy, while the addition of Ag weakens its corrosion resistance. This is in consistence with the results shown above. The weight‐averaged ε_M–M_ and ε_M–O_ of these alloys are shown in Figure 6b. It can be seen that Ag addition leads to decreases of both ε_M–M_ and ε_M–O_, while the addition of other elements results in increases of either ε_M–M_ or ε_M–O_. These are in line with the variation of $C_{Cu}^{BD}$ when the elements are added to Cu–Zr system, further confirming that selection of alloying element can be guided by ε_M–M_ and ε_M–O_ of the element and the interatomic interactions can be reflected by the weight‐averaged ε_M–M_ and ε_M–O_.

**Figure 7:**
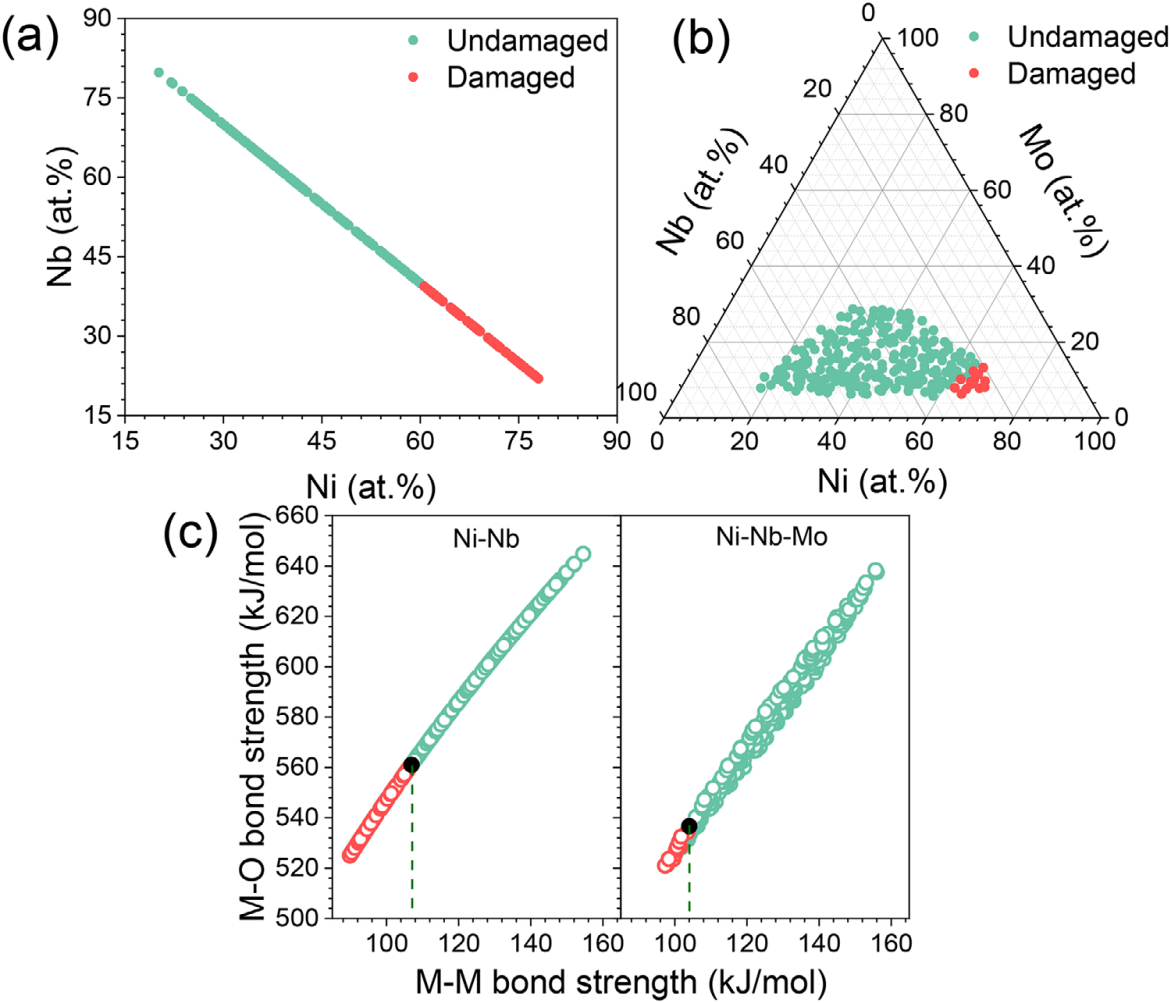
Example of identifying the element characteristics on corrosion behavior through the Ni–Nb–(Mo) alloy systems. a) Compositional characterization for Ni–Nb alloy library after immersion 5 h in a solution containing 3.5 wt.% NaCl with 2 mol L^−1^ H_2_SO_4_ at 50 °C, the red dots represent the damaged alloys after immersion, while the green dots represent the undamaged alloys. b) The same for Ni–Nb–Mo alloy system. c) The average ε_M–M_ and ε_M–O_ for 498 alloys from Ni–Nb and Ni–Nb–Mo alloy systems. The black dots indicate the threshold ε_M–M_ and ε_M–O_ of undamaged alloy thin film in the corresponding alloy systems.

To ultimately test the effectiveness of ε_M–M_ and ε_M–O_ guided selection of alloying elements for enhanced corrosion resistance, we use Ni–Nb as the base alloy system. The ε_M–M_ values for Ni and Nb are 71.3 and 182.5 kJ mol^−1^, respectively. The ε_M–O_ for Ni and Nb are 457, 686.5 kJ mol^−1^, respectively.^[^ ^13^ ^]^ Because both the ε_M–M_ and ε_M–O_ of Ni are lower than those of Nb, Ni is preferentially dissolved during corrosion. From the consideration of ε_M–M_ and ε_M–O_, the dissolution of Ni can be suppressed by alloying Ni‐Nb alloys with elements of higher ε_M‐M_ and ε_M‐O_. We therefore introduce Mo into Ni–Nb alloy system because both ε_M–M_ (164.5 kJ mol^−1^) and ε_M–O_ (608 kJ mol^−1^) of Mo are higher than that of Ni element. **Figure** **7**a,b show the plots displaying the damaged and undamaged alloys for the binary Ni–Nb alloy library and ternary Ni–Nb–Mo alloy library after 5‐h‐immersion in mixed solutions containing 3.5 wt.% NaCl and 2 mol L^−1^ H_2_SO_4_. It turns out that the corrosion‐induced film damage is primarily in the Ni–rich region of alloy library, consistent with the low ε_M–M_ and ε_M–O_ of Ni. Chemical analysis at the boundary of damaged and undamaged regions revealed that $C_{Ni}^{BD}$ is 61.1 ± 1.7% for the Ni–Nb system and 62.5 ± 1.4% for the Ni–Nb–Mo system. The increase of $C_{Ni}^{BD}$ indicates an enhanced corrosion resistance due to the introduction of Mo, confirming the ε_M–M_ and ε_M–O_ guided selection rule. Figure 6c displays the values of ε_M–M_ and ε_M–O_ for the two alloy libraries. The critical values of ε_M–M_ and ε_M–O_ at $C_{Ni}^{BD}$ are 106.9 and 561.1 kJ mol^−1^ for the Ni–Nb alloy system. The values decrease to 104 and 536.6 kJ mol^−1^ in the Ni–Nb–Mo alloy system. This suggests that the introduction of Mo allows the alloy to maintain its resistance to corrosion at higher Ni concentration, a similar phenomenon observed in the Cu–Zr–X alloy systems. It is interesting to note that the solution for corrosion tests of Ni–Nb–Mo is different from that for Cu–Zr–X alloys. However, a similar trend is observed. These findings demonstrate that the ε_M–M_ and ε_M–O_ guided selection of alloying elements is applicable in different alloy systems and environments.

In previous research, the exploration of alloys with excellent corrosion resistance typically involved a series of steps, including raw material preparation, individual alloy fabrication, structural characterization, and performance testing.^[^ ^22^, ^23^, ^30^, ^41^ ^]^ Traditional methods for evaluating the corrosion resistance of alloys mainly rely on electrochemical corrosion measurements and analyses of electrochemical impedance spectra or passive film.^[^ ^42^ ^]^ While these methods can provide in‐depth insights into the corrosion mechanisms of specific alloys, developing alloys with outstanding corrosion resistance within a broad compositional space remains challenging. Our work using a combinatorial approach reveals the intrinsic correlation between the element characteristics and the corrosion behavior of alloys through straightforward immersion experiments. For Cu–Zr base alloy system, both the ε_M–M_ and ε_M–O_ values for Cu is lower, thus the Cu–rich regions of the libraries are prone to be corroded. The addition of element with high ε_M–M_ and ε_M–O_ values than Cu element increases the activation energy barrier for its dissolution from the matrix and promotes the formation of passive films to inhibit its dissolution and thus enhance corrosion resistance. The ε_M–M_ and ε_M–O_ guided selection of alloying element is confirmed not only in the Cu–Zr system but also in the Ni–Nb system, implying its universal role in the design of corrosion‐resistant alloys. For instance, the rule can explain the enhanced corrosion resistance in the Fe–Cr–Ni alloy system. In this system, reducing the Ni content and increasing the Fe content enhances the pitting potential of the alloy.^[^ ^43^ ^]^ This can be ascribed to the larger ε_M–M_ and ε_M–O_ values of Fe than that of Ni. The increase of Fe and decrease of Ni increase both ε_M–M_ and ε_M–O_, thereby improving corrosion resistance. Our work confirms the role of ε_M–M_ and ε_M–O_ in affecting corrosion resistance and indicates that they can be used as fundamental descriptors in predicting corrosion‐resistant alloys by machine learning approaches. In this sense, our work represents an enormous step forward in guiding the design of corrosion‐resistant alloys, in particular in the case where the dominant parameters or descriptors are far from clearly identified.^[^ ^15^, ^44^, ^45^, ^46^ ^]^ It should also be noted that our current study mainly explores the influence of alloying elements on the corrosion performance of alloys from the perspective of atomic bond strength. The quantitative mechanism requires further investigation.

## 3 Conclusion

In summary, by employing a straightforward yet effective high‐throughput characterization approach, we collected corrosion resistance data for 1872 alloys across 8 alloy systems primarily in 3.5 wt.% NaCl solution. The data enabled us to analyze how the addition of specific alloying elements to a binary alloy system alters the critical alloy concentration required for corrosion resistance under certain conditions. The alteration reflects the effect of alloying elements on corrosion resistance. It was found that the effect is strongly correlated with the strength of metal–metal bond (ε_M–M_) and the metal–oxygen bond (ε_M–O_). This correlation confirms the early proposal by Marcus that elements of high ε_M–M_ and ε_M–O_ is beneficial for enhanced corrosion resistance. Furthermore, from the perspective of interatomic interactions, it was revealed that the effect of adding elements of high ε_M–M_ and ε_M–O_ to a base alloy system is in fact to lower the critical weight‐averaged ε_M–M_ and ε_M–O_ required for corrosion resistance. The findings about the roles of ε_M–M_ and ε_M–O_ may shift our focus in developing corrosion‐resistant alloys from the traditional trial‐and‐error approach to the intrinsic characteristics of the constituent elements of alloys. In addition, the findings bear importance for the construction of an accurate machine‐learning model and data‐driven design of corrosion‐resistant alloys.

## 4 Experimental Section

### Compositional Libraries Preparation

The combinatorial thin film libraries of Cu–Zr–X (X: Ag, Cr, Al, Ti, Ta) and Ni–Nb–(Mo) were fabricated by confocal DC‐magnetron co‐sputtering (Ace Precision Machine, Inc., AFS1800) deposition at a deposition rate of ≈8 nm min^−1^ from elemental sputtering targets with purity better than 99.95%. The sputtering power ranges from 7 to 14 W for the Cu target, 36 to 47 W for the Zr target. The sputtering powers for the Ag, Cr, Al, Ti, Ta, Ni, Nb, and Mo targets are 5, 13, 20, 27, 10, 16, 37, and 8 W, respectively. The 100‐mm‐diameter single‐side‐polished Si wafers were used as substrates. The base pressure was lower than 8×10^−5^ Pa, and the working pressure was 0.9 Pa. For each compositional library, we fabricated 229 compositions with a diameter of ≈3.6 mm. The spacing between the compositions is 5 mm.

### Materials Compositions and Structure Characterizations

The chemical compositional of the Cu–Zr–X and Ni–Nb‐(Mo) combinatorial films were automatically measured by energy dispersive X‐ray spectrum (EDX) attached to a Phenom scanning electron microscope (SEM). The structure of the libraries was characterized by using a Malvern PANalytical Empyrean X‐ray diffractometer with a Cu Kα radiation source, which enabled the rapid X‐ray diffraction (XRD) mapping.

### Immersion Testing

The as‐prepared combinatorial thin film libraries were individually immersed in Petri dishes containing 3.5 wt.% NaCl aqueous solution (≈200 mL) at 25 °C. After 320 min of immersion, the samples were taken out of the solution and cleaned with deionized water to remove surface residues for further characterization.

### Spectroscopic Ellipsometry Testing

The Spectroscopic ellipsometry (SE) (SE‐VE) experiments were carried out to measure the optical constants of combinatorial libraries at a fixed angle of incidence of 65° in air atmosphere. The range of wavelength is from 400 to 800 nm. The SE measurement at each point encompassed three sequential components, focusing, measuring, and curve fitting. The optical properties of reflectivity of the samples were obtained by modeled with B‐spline method.^[^ ^25^ ^]^

## Conflict of Interest

The authors declare no conflict of interest.

## Data Availability

The authors declare that the data supporting the findings of this study are included in the paper.
